# Sparse and Expandable Network for Google's Pathways

**DOI:** 10.3389/fdata.2024.1348030

**Published:** 2024-08-29

**Authors:** Charles X. Ling, Ganyu Wang, Boyu Wang

**Affiliations:** Department of Computer Science, Western University, London, ON, Canada

**Keywords:** continual learning, lifelong learning, few-shot learning, catastrophic forgetting, sparsity

## Abstract

**Introduction:**

Recently, Google introduced Pathways as its next-generation AI architecture. Pathways must address three critical challenges: learning one general model for several continuous tasks, ensuring tasks can leverage each other without forgetting old tasks, and learning from multi-modal data such as images and audio. Additionally, Pathways must maintain sparsity in both learning and deployment. Current lifelong multi-task learning approaches are inadequate in addressing these challenges.

**Methods:**

To address these challenges, we propose SEN, a Sparse and Expandable Network. SEN is designed to handle multiple tasks concurrently by maintaining sparsity and enabling expansion when new tasks are introduced. The network leverages multi-modal data, integrating information from different sources while preventing interference between tasks.

**Results:**

The proposed SEN model demonstrates significant improvements in multi-task learning, successfully managing task interference and forgetting. It effectively integrates data from various modalities and maintains efficiency through sparsity during both the learning and deployment phases.

**Discussion:**

SEN offers a straightforward yet effective solution to the limitations of current lifelong multi-task learning methods. By addressing the challenges identified in the Pathways architecture, SEN provides a promising approach for developing AI systems capable of learning and adapting over time without sacrificing performance or efficiency.

## 1 Introduction

The past decade has seen significant growth in the capabilities of artificial intelligence. Deep learning in particular has archived great successes in medical image recognition and diagnostics (Litjens et al., 2017; Shen et al., 2017), tasks on natural language processing (Devlin et al., 2019; Radford et al., 2019), and learning to play difficult games at the expert level (Silver et al., 2017). However, as pointed out recently by Google (Dean, 2023), current deep learning models are often trained from scratch for each new task. The individually trained models cannot leverage each other to improve their performance. If the same model is trained on additional tasks in sequence, the previous ones would likely be *forgotten* and their performances can degrade greatly.

Several lines of research have been published to overcome these weaknesses. Multi-task learning (Caruana, 1997) considers how to learn multiple tasks and concepts at the same time such that they can leverage the knowledge learned from each other. The related field of transfer learning (Pan and Yang, 2009) assumes that some tasks have been previously learned and aim to transfer their knowledge assisting in learning new tasks. Few-shot learning (Fei-Fei et al., 2006) focuses on the scenario where only a small number of labeled data are available. Lifelong learning (Thrun., 1995; Thrun, 1998), also known as continual (Parisi et al., 2019) or sequential learning (McCloskey and Cohen, 1989), considers how to learn and transfer knowledge across long sequences of tasks without catastrophic forgetting. More recently, Peng and Risteski (2022) presents a formalization of continual learning through feature extraction, proposing an efficient gradient-based algorithm, DPGrad, for linear features that maintains performance across environments without catastrophic forgetting, and demonstrates that similar approaches for non-linear features face fundamental limitations. Wang et al. (2022) introduces SparCL, a novel framework for efficient continual learning on edge devices that enhances training speed and accuracy through task-aware dynamic masking, dynamic data removal, and dynamic gradient masking, significantly improving upon existing methods in resource-limited scenarios.

However, most previous approaches can only demonstrate subsets of the properties of multi-task and lifelong learning often by different complex mechanisms. For example, existing lifelong learning techniques tend to use one or more of three types of mechanisms, each of which comes with its own drawbacks and hurdles (De Lange et al., 2019). These mechanisms are respectively based on replay, regularization, and dynamic architecture. See Section 2 for a brief review. It would be extremely useful if we could have *one* general deep learning model that can learn new tasks continuously without forgetting and can leverage and transfer knowledge between tasks in improving their learning.

This is exactly what was proposed recently by Google (Dean, 2023) to construct the so-called Pathways as its next-generation AI architecture. Google's Pathways is one general model for learning many tasks continuously without catastrophic forgetting and with knowledge transfer between tasks. Pathways also need to be sparse, and able to handle multi-modality of data. Though posted as a blog, these requirements have long been recognized as challenges for machine learning and AI.

We first describe the three challenges of Google's Pathways in more detail. We add our own formulation to make them more clear.

### 1.1 Challenge 1: continual learning of one general model for many tasks

In Google's Pathways, it is assumed that the tasks {(*T*_1_, *D*_1_), (*T*_2_, *D*_2_), ...} are given in sequence, where *D*_*i*_ is the data of task *T*_*i*_. Instead of constructing separate models for each task, *T*_*i*_, one general (deep learning) model must be built by increasingly introducing more new tasks. However, the model must be able to provide reliable predictions at any time for previous tasks; thus, one cannot trivially wait to gather all data of all tasks to build one deep learning model at the end. It is also required that when the new task *T*_*N*_ is learned from *D*_*N*_, the data of previous tasks (i.e., {_*D*_*i*_}*i* ≤ *N*−1_) should not be used.

Assuming *T*_1_, *T*_2_, ..., *T*_*N*_ have been learned, Pathways must exhibit several fundamental properties of lifelong learning when learning a new task *T*_*N*+1_:

- No catastrophic forgetting: this is the ability to avoid a dramatic loss in performance on *T*_*i*_ (*i* ≤ *N*) when learning *T*_*N*+1_ from *D*_*N*+1_ (McCloskey and Cohen, 1989).
- Forward transfer: This is the ability to learn a new task *T*_*N*+1_ easier (with fewer training examples to achieve the same or higher predictive performance) by leveraging the knowledge from earlier learned tasks {_*T*_*i*_}*i* ≤ *N*_. This is also known as knowledge transfer (Pan and Yang, 2009). Achieving sufficient positive forward transfer makes it possible for few-shot learning on the new tasks.
- Backward transfer: this is knowledge transfer from *T*_*j*_ to *T*_*i*_ where *i*<*j*, the opposite direction as forward transfer. When learning a task *T*_*j*_ after *T*_*i*_, *T*_*j*_ may in turn help to improve the performance of an earlier talk *T*_*i*_. This is like a “review” before a final exam after the materials of all chapters have been taught and learned. Later materials can often help better understand earlier materials.

### 1.2 Challenge 2: learning tasks with multiple modalities

As outlined in Dean (2023), Pathways should enable multi-modality models that encompass vision, auditory, and language understanding simultaneously. “So whether the model is processing the word ‘leopard', the sound of someone saying “leopard” or a video of a leopard running the same response is activated internally: the concept of a leopard.” Most current deep-learning models cannot easily handle data from multiple modalities.

### 1.3 Challenge 3: sparsity in learning and predicting

Another major issue pointed out in Dean (2023) is that most current deep learning models are *dense*, which means the whole neural network would activate to accomplish a task. A dense network is certainly very computationally and energy inefficient, especially for very large models.

Although no formal measurement of the sparsity is given, we use the percentage of the network weights that are activated during training and testing as an indication of the network sparsity.

### 1.4 Contributions

In this paper, we propose SEN, Sparse and Expandable Network, which utilizes and integrates with several previous works in a novel way. SEN is actually simple, yet it effectively answers the three challenges of Google's Pathways. The main contributions of our work are:

1. We introduce a novel *task dispatcher*, a classifier that learns from the task data and makes the whole network sparse. The dispatcher can also exploit the relevance of the tasks, and fulfill the knowledge transfer among them in an effective fashion. See Section 3.1.
2. When learning a new task, SEN freezes the current network so forgetting of previous tasks will not happen. It then expands the network to learn the new task, which can be in a different modality. We combine the ideas of weight regularization (Kirkpatrick et al., 2016; Li and Hoiem, 2017; Zenke et al., 2017; Chaudhry et al., 2018; Ritter et al., 2018; Zhang et al., 2020) and dynamic architecture (Rusu et al., 2016; Xu and Zhu, 2018; Yoon et al., 2018) with our modifications. See Section 3.2 for details.
3. After the dispatcher finds relevant tasks that can be leveraged, forward and/or backward transfer links between tasks are enabled and initiated, ready to be learned from the data. Thus, only the weights of the relevant tasks are trained and updated. In this way, the knowledge between tasks is transferred yet the activation in the network is still sparse.
4. Though our experiments are relatively small in scale, we demonstrate that SEN can, in principle, effectively meet all of the three challenges in Google's Pathways. SEN also outperforms baseline models by a wide margin with small training datasets and exhibits many important properties of lifelong multi-task learning.

## 2 Related works

Previous work on lifelong machine learning and continual learning tend to fall into three categories, and they can often only demonstrate subsets of properties proposed in Google's Pathways. The first category, replay (or generative replay), commonly works by storing (or generating) previous task data and training on it alongside new task data (Rebuffi et al., 2017; Isele and Cosgun, 2018; Chaudhry et al., 2019; Wu et al., 2019). As a result, its data and computation efficiency is prohibitively low for the Pathways.

The second category is regularization. This mechanism, examplified by Elastic Weight Consolidation (EWC), works by restricting weight changes (making them less “flexible”) so that learning new tasks does not significantly affect previous task performance (Kirkpatrick et al., 2016; Li and Hoiem, 2017; Zenke et al., 2017; Chaudhry et al., 2018; Ritter et al., 2018; Zhang et al., 2020; Yang et al., 2023). We use a special version of the weight regularization in our model SEN. More specifically, we propose to use weight regularization more strategically by simply freezing weights for all non-forgetting tasks. This eliminates forgetting while also reducing network sparsity and computation on weight updating. Note however, we do allow relevant tasks to leverage each other by initiating trainable weights for forward and backward knowledge transfer.

The third category is dynamic architecture (Rusu et al., 2016; Xu and Zhu, 2018; Yoon et al., 2018; Kang et al., 2022). They commonly work by adding new units and weights for each task and may only allow parts of the weights to be tuned. This reduces forgetting while also allowing knowledge transfer. However, without a task dispatcher as in our model, the network activity is always at 100%. In our model, the network architecture is dynamically expanded, and with the dispatcher and selective forward and backward transfer links, we can efficiently achieve both sparsity and knowledge transfer within the network.

Very recently Google published a system-level design for asynchronous distributed dataflow for the Pathways (Barham et al., 2022). However, it does not deal with machine learning tasks specifically. There are no experiments or discussions on how continual and multi-task learning, non-forgetting, forward and backward transfer, and sparsity are achieved. Google further introduces a novel routing algorithm called Expert Choice (EC) (Zhou et al., 2022) that addresses load imbalance and under-utilization in mixture-of-experts (MoE) models, achieving significant improvements in training efficiency and downstream performance compared to traditional methods.

## 3 Sparse and Expandable Network

In this section, we propose a Sparse and Expandable Network (SEN), which is quite simple yet effective in meeting the basic challenges of Google's Pathways.

Google's original Pathways description misses one crucial component: when a new task needs to be predicted by the Pathways, which input model or models should it be sent to? Some previous work assumes that this information is given (Rusu et al., 2016; Li and Hoiem, 2017; Mallya and Lazebnik, 2018). This assumption is certainly not realistic for Pathways, as Pathways itself needs to decide which model(s) the input task should be sent to.

**Figure 1:**
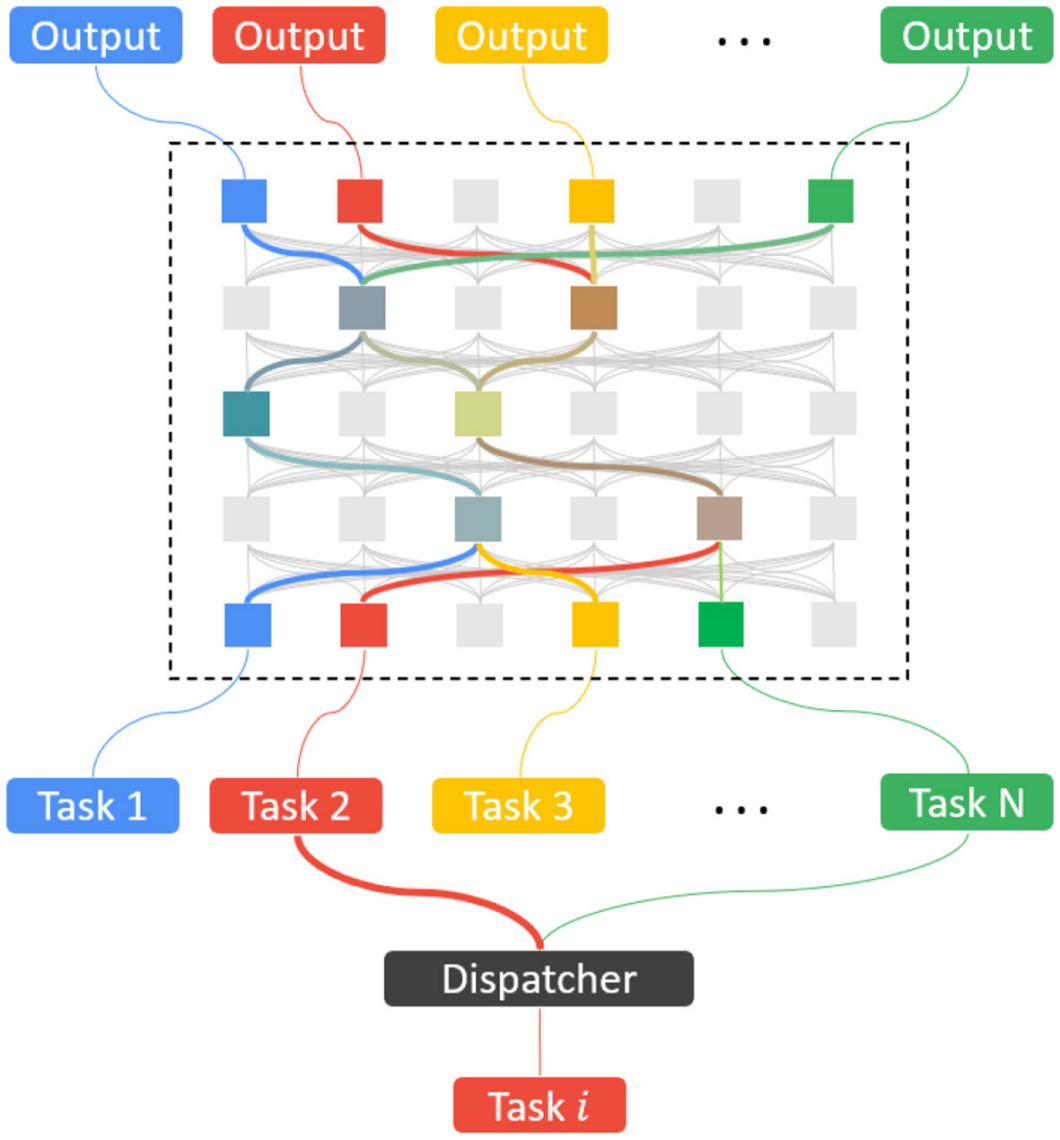
The SEN framework with the dispatcher classifier.

In our model SEN, we add this crucial component, a dispatcher classifier or “job router,” which itself is learned progressively from the data of the tasks. The dispatcher will identify and activate a small number of task-specific models so that the whole network is sparse in its activity. See Figure 1 for the SEN model with the dispatcher.

### 3.1 Dispatcher classifier

The dispatcher classifier is trained from the data *D*_1_, *D*_2_, ..., *D*_*N*_ of the related tasks *T*_1_, *T*_2_, ..., *T*_*N*_ in the continual learning fashion. The process is the same as learning a new task as in Section 3.2. As an example, assume that *T*_1_ is a 10-way classification of the Fashion-MNIST (Xiao et al., 2017), and *T*_2_ is a 10-way classification of the MNIST. If the new task *T*_3_ is to classify 26 hand-written letters from “a” to “z,” then the dispatcher learns a three-way image classification of Fashion, MNIST, and lower letters, using the corresponding training data. For the new task *T*_3_, a new binary classification model for *T*_3_ is learned from the data of *T*_3_, and it becomes a part of the dispatcher. If there are both image and audio inputs, the dispatcher may directly classify them by the data type of the input data.

### 3.2 Learning new tasks in SEN

**Figure 2:**
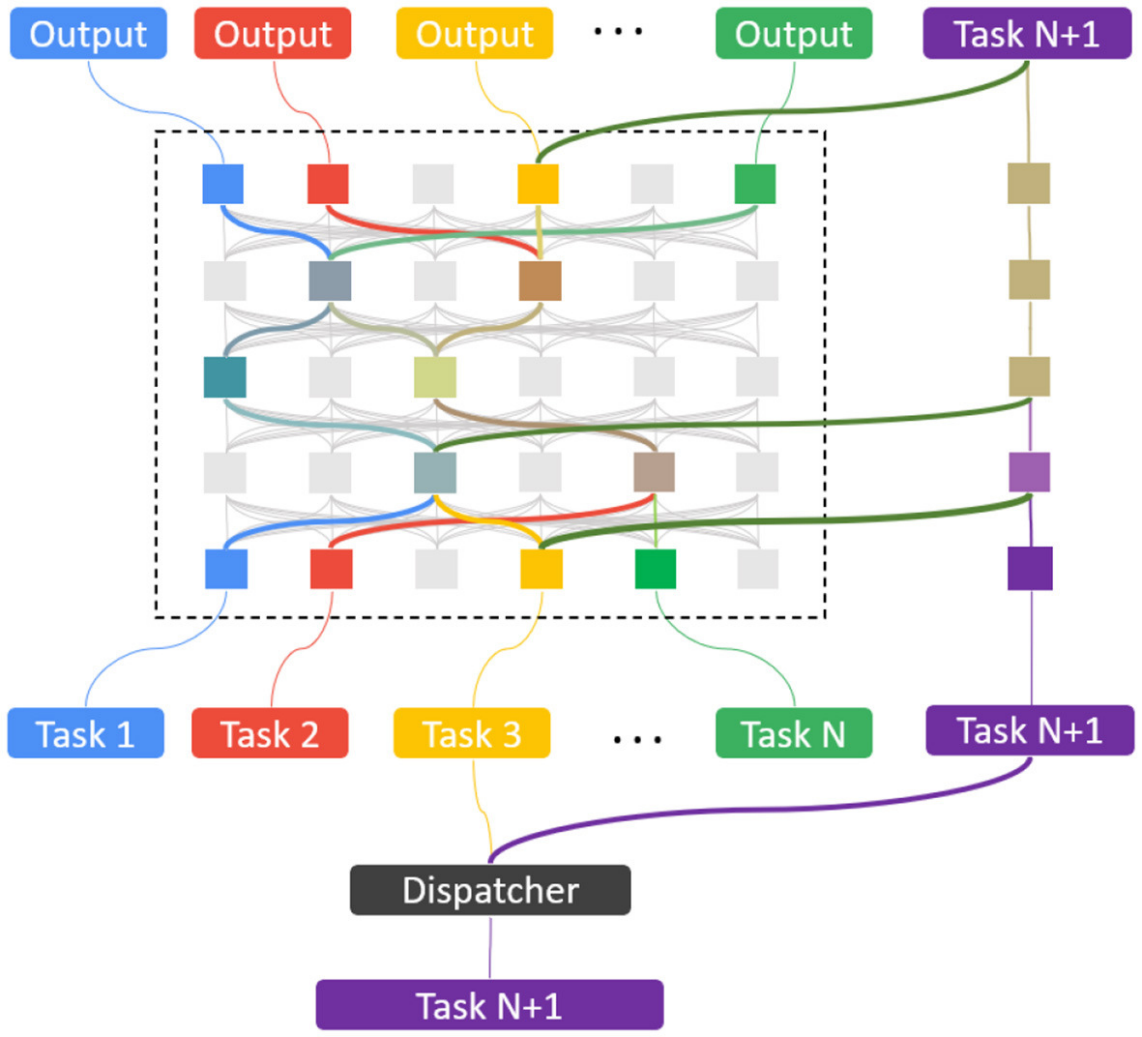
Learning a new task in the SEN framework.

Figure 2 illustrates how SEN is expanded and learned with the new tasks without forgetting previous tasks. The pseudo-code in Algorithm 1 describes how to learn a new task *T*_*N*+1_ in the SEN framework after previous tasks *T*_1_, ...*T*_*N*_ have been learned.

**Algorithm 1:**
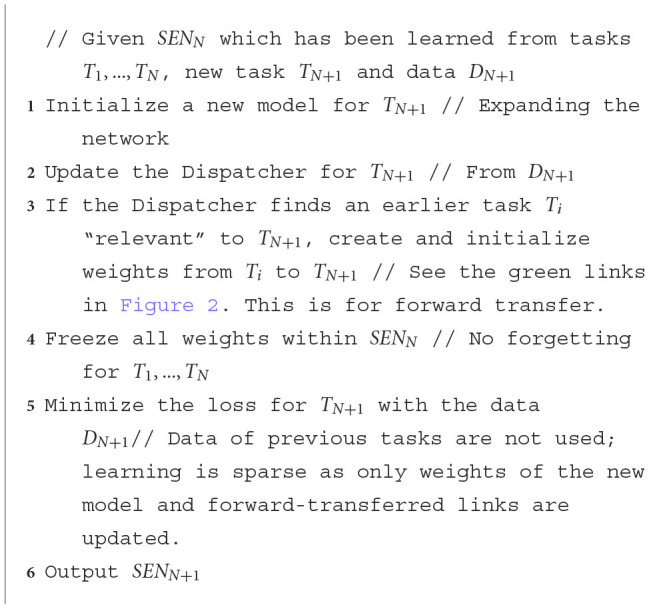
Continual Learning of new tasks in SEN.

Non-forgetting is guaranteed by construction with weights of the previous tasks *T*_1_, ..., *T*_*N*_ frozen while training the new pathway for the new task *T*_*N*+1_. Sparsity is also guaranteed by the dispatcher; it only activates a small number of task models during training and testing, making the network computationally efficient.

### 3.3 Forward and backward knowledge transfer

As we have seen in Figure 2, when the Dispatcher finds an earlier task *T*_*i*_ *relevant* to new task *T*_*N*+1_, forward transfer links from *T*_*i*_ to *T*_*N*+1_ will be created and initialized, ready to be trained by minimizing the loss function on *T*_*N*+1_. The *relevancy* of *T*_*i*_ and *T*_*N*+1_ is determined by the similarity of the two tasks; that is, similar tasks can assist each other in both forward and backward transfer. This is analogous to real-life human learning. For example, driving a car can assist in learning to drive a truck as they are similar, but usually not for cooking steaks as driving and cooking are very different.

In our work, the similarity is measured by how well (with a predefined threshold, 0.7 in SEN) the previous task model can classify a subclass of the new task data. Let us use a concrete example to explain this. If *T*_*i*_ is the 10-way classification of MNIST digits, and *T*_*N*+1_ is the 26-way classification of handwritten capital English letters. Then, when training data of the class “O” in *T*_*N*+1_ are presented to the dispatcher, it may predict that it can be *T*_*i*_ (with a probability over the threshold 0.7), because *T*_*i*_ has a class “0” (the digit zero), which is highly similar to “O.” The links from task “0” to “O” are initiated as forward transfer links, so that the representation learned in “0” can be leveraged in learning “O.” In particular, the output layer weights of “0” can be forward-transferred and initiated to be the output weights of “O.” In this case, the training of new class “O” in *T*_*N*+1_ would achieve higher accuracy compared to without such forward transfer links, even with a few training examples of “O.” Similar cases can happen between “1” and “I,” “2” and “Z.” Of course this does not always happen as the similarity is determined by the particular training data and the threshold. See our Experiment section for details.

Likewise, backward transfer can happen from *T*_*j*_ to *T*_*i*_ (*i*<*j*) when *T*_*j*_ and *T*_*i*_ are similar enough. Backward-transfer inks from *T*_*j*_ to *T*_*i*_ will be created and imitated, and *T*_*i*_ is fine-tuned (or re-trained) with the links from *T*_*j*_. This can improve the accuracy of *T*_*i*_ with the knowledge of *T*_*j*_. Note that during training of *T*_*i*_, weights of all other tasks will be frozen to prevent forgetting of other tasks.

### 3.4 Learning with multi-modality data

One major challenge posed in Dean (2023) and for current machine learning models is that learning from multi-modality data, such as vision, auditory, and language understanding, cannot be handled simultaneously and effectively.

**Figure 3:**
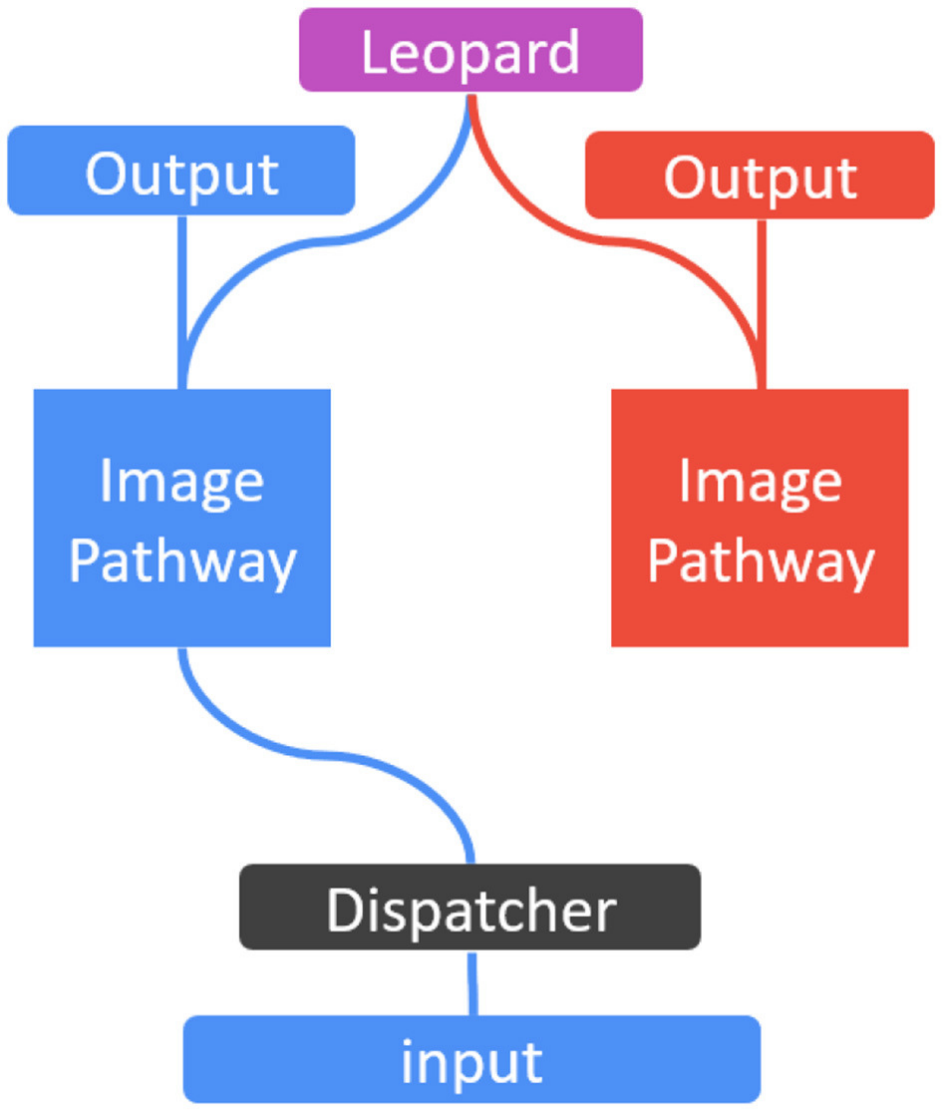
An illustration of SEN for multi-modality tasks.

Learning with multi-modality data can be easily implemented in SEN. This is because SEN creates a new task-specific model for each new task, and the new model can be a CNN, LSTM, or even SVM or random forests. Figure 3 shows such a multi-modality SEN, with one pathway for image classification of animals including the leopard, and another pathway for audio classification of words including “leopard.” These pathways are trained simultaneously by images and audio signals when the image of “leopard” is shown and the word “leopard” is spoken. As these are two different data types (i.e., images and audio), the dispatcher can easily classify them, and send the right training data to the right pathways. In the output nodes, the label “leopard” is shared in both tasks of image and audio classifications, and the training can happen in the image and audio pathways in parallel. This is essentially multi-modality learning studied previously (Nawaz et al., 2019; Gallo et al., 2020).

In our experiments, we use MNIST digits and EMNIST letters for the imaging modality, and Audio-MNIST (Jackson, 2016) of spoken digits as the audio modality to conduct our experiments.

### 3.5 Sparsity of SEN

As we have seen, during training in SEN, we choose to transfer from at most one previous task to learn the new task. To maintain sparsity during testing, the dispatcher also only selects one or two most likely pathways to activate. Assume that a total of *N* tasks have been learned in SEN. As SEN only activates at most two pathways for the learning and testing, only 2/*N* of the whole network weights are activated at any phase, assuming each task model consumes the same number of weights. When *N* is large, such as 1,000, at most 0.2% of the weights are activated. This allows SEN to maintain sparsity yet still facilitate knowledge transfer between task models.

Clearly, if without any forward and backward transfer between task models, tasks would be trained individually, which must be avoided. On the other extreme, if we allow a task model to transfer back and forth to all other task models, the whole network would become a fully connected dense network, in which the sparsity cannot be achieved. Thus, a good trade-off balance between sparsity and the amount of knowledge transfer is needed in SEN by the setting of model parameters (e.g., the number of tasks in knowledge transfer, which is 1, and the similarity threshold, which is 0.7, in our work).

### 3.6 Network pruning and graceful forgetting in SEN

One potential concern with SEN is that the total size of the network grows linearly with the number of tasks. There are two popular approaches to alleviate this issue. The first one is network pruning, studied previously (Mallya and Lazebnik, 2018), to free up units in each task model without affecting much its performance. The second approach is the so-called *graceful forgetting*. Instead of freezing the weights of previous tasks, the weights of some previous tasks can be allowed to be trained with the new task. This results in gradual forgetting (or deterioration) of the performance of the previous tasks but it allows the new task to be learned with fewer new units (Bohn et al., 2021).

Notice that even if SEN may become large as the number of tasks grows, the activation in SEN is always sparse, as we have discussed.

## 4 Experiments

In this section, we will describe the experimental results of the comparison with SEN and other baseline models on learning a sequence of classification tasks with different modalities.

### 4.1 Sequence of tasks

In the experiments, five classification tasks are selected as the sequence of learning tasks. We utilize a task-incremental setting, where the task index is provided to the model. These five tasks are:

Task 1: Fashion-MNIST (Xiao et al., 2017). A 10-way classification of gray-scale images of fashion clothes, with the image size 28 by 28. It contains a training set of 60,000 and a testing set of 10,000 images. As we will compare different models with few-shot learning, random sampling from the original training set is performed to form our own training set.

Task 2: MNIST (Deng, 2012). A dataset of gray-scale images of hand-writing digits from “0” to “9.” The size of each image is also 28 by 28. It contains 60,000 training images and 10,000 testing images.

Task 3: LOWER. This is to learn to classify 26 hand-written lowercase English letters from “a” to “z.” It is extracted from EMNIST (Cohen et al., 2017), thus each image is also 28 by 28 gray-scaled. This task contains 163,939 images of lowercase letters.

Task 4: UPPER. This is similar to Task 3 except it consists of 26 classes of hand-written uppercase letters from “A” to “Z.” This task contains 188,958 images of uppercase letters extracted from the EMNIST dataset.

Task 5: Audio-MNIST. Free Spoken Digit Dataset (FSDD) (Jackson, 2016) is a dataset with a collection of spoken digits, containing 2,500 recordings of 10 digits from five speakers (50 samples of each digit per speaker). This is used as Task 5 for testing the multi-model ability of the SEN.

As Task 1 to Task 4 are all gray-scale images with the same size, the dispatcher must learn to distinguish and classify them with task data. Task 5 has a completely different representation thus the dispatcher can distinguish images from audio from data types.

### 4.2 SEN and baseline methods

For image models in SEN, we use simple two-layer MLP (Multilayer perceptron) for Tasks 1 to 4. There are 128 units in the first layer and the activation function is ReLU. The size of the second layer is adapted to the task; that is, the number of units in this layer depends on the number of classes in the task. The activation function is Softmax. Note that although the SEN's task models for images are very simple, we use the same MLP base model in comparing other baseline methods.

Task 5 is Audio-MNIST. We first pad the data to the same length and convert it to Mel spectrum with the number of Mel spectrum of 64. The model for this task is an LSTM of 500 units, with an average on the time step axis, followed by three-layer MLP with 128, 64, and 10 units. The first two layers use ReLU as the activation function and the last layer uses the Softmax activation function.

Several baselines for comparison are:

- Multi-task (seq): a non-expanding multi-task model which learns all tasks sequentially. It is expected that forgetting previous tasks would occur, and forward transfer is weak.
- Single-task: single-task models which are separate and trained by individual tasks. It is expected that forgetting will not happen, but no knowledge will be transferred between models.
- PNN: Progressive Neural Network proposed in Rusu et al. (2016), with the same base model as SEN. PNN uses dynamic architecture for lifelong multi-task learning.
- EWC: Elastic Weight Consolidation, a regularization method proposed in Serrà et al. (2018), with the same number of hidden units as the other baseline models.

### 4.3 Catastrophic forgetting

**Figure 4:**
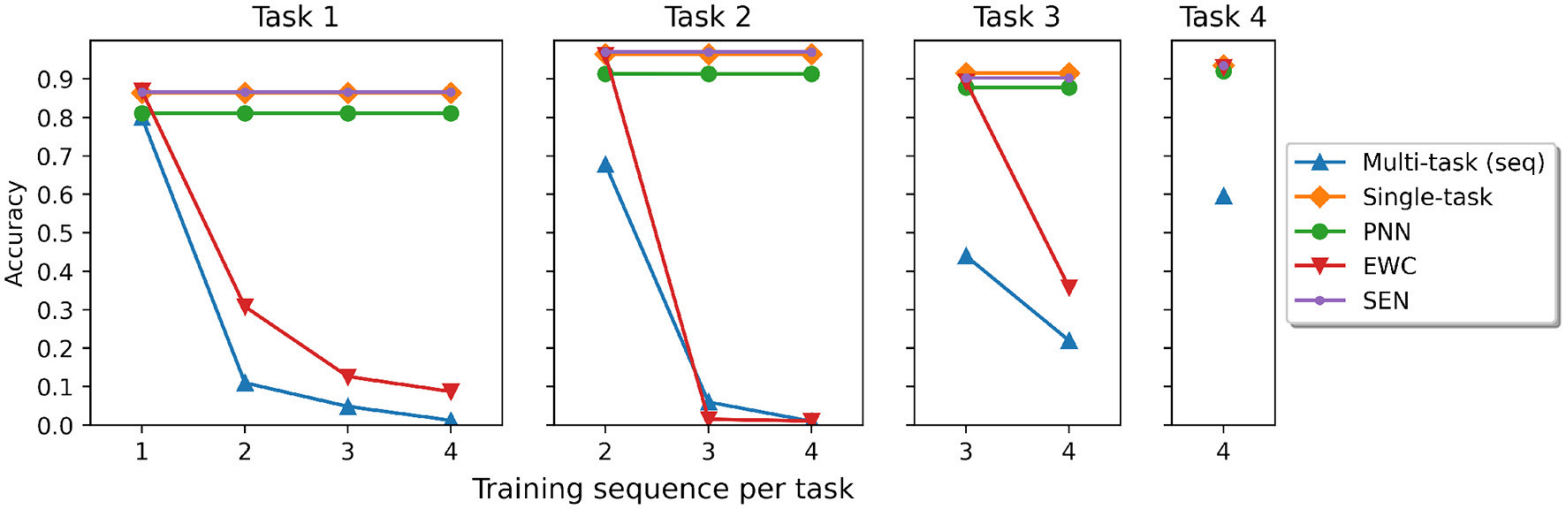
The accuracy for training four tasks sequentially. Note that Task 5 is audio-MNIST and is not trainable in other models. SEN can train Task 5 with an independent LSTM model, thus no forgetting happens in the previous tasks.

We train SEN and other baselines with the five tasks sequentially. Figure 4 shows the predictive accuracy for each task when the model is sequentially trained with more tasks. The result clearly shows that forgetting does not happen in SEN, PNN, and Single networks. This is expected as non-forgetting is inherent when constructing the models. We can also see that catastrophic forgetting still happens in EWC, and is most severe in the multi-task (seq) models.

### 4.4 Knowledge transfer between tasks

When learning new tasks, SEN may detect a previous similar task, and transfer the relevant knowledge to the new task, as described in Section 3.2. Table 1 shows the details of such forward transfer from a previous task to a new one. Tasks 1 and 2 (Fashion-MNIST and MNIST) are the first two tasks. As they are quite dissimilar, no transfer occurs.

**Table 1:** Details of knowledge transfer between tasks.

	**Task 1 (Fashion)**	**Task 2 (MNIST)**	**Task 3 (LOWER)**	**Task 4 (UPPER)**	**Task 5 (Audio)**
Transfer details	No transfer	No transfer	0 → o: 0.936	z → Z: 0.912	No transfer
			1 → l: 0.907	s → S: 0.897	
			5 → s: 0.840	4 → H: 0.857	
			2 → z: 0.764	o → O: 0.855	
				t → T: 0.855	
				v → V: 0.838	
				m → M: 0.837	
				f → F: 0.836	
				u → U: 0.821	
				x → X: 0.815	
				c → C: 0.768	
				w → W: 0.753	
				k → K: 0.752	
				0 → D: 0.743	
				p → P: 0.735	
				1 → I: 0.715	
				l → I: 0.709	

The number is the probability that the target class is predicted as the source class in the source pathway, which indicates the similarity between the source task and the target class. In our work, we set the similarity threshold to be 0.7.

When learning Task 3 (lowercase letters), we see transfers happen from Task 2, though not every transfer is desired or robust, as the similarity is detected from the training data. For example, “5” in MNIST is transferred to “s” and “2” is transferred to “z.” We set the similarity threshold to be 0.7.

The most interesting and powerful transfer occurs in learning Task 4 (upper case letters). Many reasonable transfers from Task 3, and a few from Task 2, are observed, as many lowercase letters are similar to uppercase letters. This kind of knowledge transfer assists in learning of new tasks, improving predictive accuracy even with a small number of training data.

The comparison of the predictive accuracy of SEN and the baselines are shown in Table 2. We can draw the following interesting conclusions:

**Table 2:** Test accuracy of SEN and baselines methods.

**Few shot-training**		**Task 1 (Fashion)**	**Task 2 (MNIST)**	**Task 3 (LOWER)**	**Task 4 (UPPER)**	**Task 5 (Audio)**
Random guessing accuracy		10.0	10.0	3.8	3.8	10.0
0-sample	Single	11.1 ± 1.7	11.2 ± 1.7	2.5 ± 2.2	3.5 ± 1.1	-
		EWC	1.1 ± 1.1	11.6 ± 1.5	3.9 ± 0.5	1.9 ± 0.5	–
		PNN	9.8 ± 2.7	8.4 ± 2.7	1.7 ± 1.2	3.7 ± 4.5	–
		SEN	9.1 ± 2.6	10.4 ± 1.6	**13.2** ± 1.8	**53.3** ± 6.1	9.9 ± 0.5
5-sample	Single	69.2 ± 0.3	63.4 ± 0.5	50.4 ± 1.0	53.5 ± 0.3	–
		EWC	66.9 ± 2.0	40.9 ± 3.2	15.3 ± 5.3	21.0 ± 3.4	–
		PNN	67.8 ± 1.4	61.1 ± 2.6	48.7 ± 1.3	51.8 ± 1.6	–
		SEN	69.4 ± 0.3	64.0 ± 0.6	50.6 ± 1.3	**69.2** ± 2.1	45.8 ± 0.2
10-sample	Single	71.4 ± 0.1	68.9 ± 0.6	56.8 ± 0.3	61.7 ± 0.6	–
		EWC	71.4 ± 0.4	54.8 ± 2.4	34.0 ± 5.8	34.1 ± 4.3	–
		PNN	71.2 ± 0.7	69.2 ± 0.1	56.1 ± 1.6	61.9 ± 0.1	–
		SEN	71.6 ± 0.4	69.0 ± 0.4	56.6 ± 1.1	**73.6** ± 1.1	57.6 ± 0.7
All-sample	Single	86.4 ± 0.9	96.5 ± 0.3	91.5 ± 2.3	93.5 ± 0.5	-
		EWC	86.9 ± 0.4	96.2 ± 0.1	89.3 ± 0.4	93.1 ± 3.8	-
		PNN	81.1 ± 1.0	91.3 ± 0.9	87.9 ± 1.2	92.1 ± 0.4	-
		SEN	87.0 ± 0.5	96.5 ± 0.4	90.5 ± 0.3	93.2 ± 0.2	86.0 ± 1.9

The bold values indicates higher score than other Algorithms.

Zero-shot learning: All baseline models perform similarly as random guesses without any training data, as expected. However, as Task 3 and especially Task 4 have leveraged from the previously learned similar tasks, their predictive accuracy by SEN, even before seeing any data, is much higher than other baselines. For Task 4, the random guessing accuracy is only 1/26 or around 3.8%. However, SEN can achieve a very high accuracy of 53.3%, due to the knowledge transfer from the lowercase letters to the uppercase letters. To make an analogy, after learning to drive cars, an adult can usually drive a truck quite well even without being trained to drive trucks, due to the similarity between cars and trucks.

Few-shot learning: With only five or 10 training examples for each class, SEN achieves much higher predictive accuracy than other baselines for Task 4, where much forward transfer has been detected and utilized. This shows the effectiveness of forward transfer, especially with a few training data. This is crucial in many real-world applications where labels are expensive to obtain. Human can often learn a new task based on a few training data.

All-sample training: SEN and other baseline methods perform similarly in all-sample training. The reason could be that, since SEN and other baseline methods use the same base models, when they are fully trained with a large dataset, all models are fully explored and acquire a similar performance.

Many previous machine-learning algorithms are trained with large-scale datasets [such as ImageNet (Deng et al., 2009)] with complex modeling and other means such as data augmentation to achieve SOTA (state-of-the-art) results. The difference of such SOTA results is often very small. Our results suggest that it would be more important to compare results under the same setting with small training datasets, and such differences can be huge. Our comparisons among different baseline methods clearly show the power of SEN when only a small amount of data is available.

### 4.5 Multi-modality data

In addition to the four tasks in the visual modality, we also introduce an audio-based classification task with the Audio-MNIST dataset as Task 5. The performances of SEN on this auditory task are also shown in Table 2. Other models cannot handle multi-modality data, but SEN has no problem of constructing an independent LSTM model for Task 5. There is no forward transfer links from image tasks to Task 5. However, if there were other audio tasks, then similarity between audio tasks may be detected, just like similarity between images of lower and upper case letters. In this case, forward transfer may assist the learning process of new audio tasks.

### 4.6 Sparsity

After all of the five tasks have been sequentially learned, we calculate the percentages of activated weights in predicting the five tasks as 6.7%, 6.7%, 13.6%, 13.8%, and 72.8% respectively. These numbers do not add up to 100% as for some tasks, two task models may be activated due to forward transfer from another task. In any case, only a small portion of SEN is activated for Tasks 1–4. If there were 1,000 tasks, each of which has the same number of weights, the model is highly sparse as only 0.1%–0.2% of the network weights are activated. Depending on how SEN is implemented, it can be more energy and computationally-efficient than most current deep learning models.

## 5 Discussions and conclusions

Google recently introduced Pathways as its next-generation AI architecture, which needs to address three major challenges simultaneously. These challenges have also been long-standing in the current lifelong multi-task learning. We propose a simple yet effective Sparse and Expandable Network (SEN) to meet these challenges. Experiments compared with other popular baseline models show the effectiveness of SEN when trained with very small training data. Much machine learning research attempts to reach or outperform SOTA trained on very large datasets using complex models and other means such as pre-training and data augmentation. The difference in such SOTA results is usually very small. Our work hopes to offer a different insight with simplicity and clarity.

In our future work, we plan to study SEN on a much larger scale with different learning tasks such as classification, regression, and reinforcement learning, with multiple data modalities such as audio, images, videos, and so on. In addition, our quantitative measure of similarity for knowledge transfer opens a way to study negative transfer. If the similarity is too low but the transfer is forced, it may do more harm than good. This is analogous to someone struggling to drive on the left side of the road after learning to drive on the right side.

## Data Availability

Publicly available datasets were analyzed in this study. This data can be found at: Fashion-MNIST (https://www.kaggle.com/datasets/zalando-research/fashionmnist), MNIST (https://www.kaggle.com/datasets/hojjatk/mnist-dataset), and Audio-MNIST (https://www.kaggle.com/discussions/general/188196).
